# Chloride of Sodium in Inflammatory Diseases

**Published:** 1884-09

**Authors:** 


### Chloride of Sodium in Inflammatory Diseases.

"Give your patients chloride of sodium," is the advice of Dr. Goss, Marietta, Georgia, and he gives the following reasons: "The fact that in severe inflammatory forms of disease salt disappears from the urine, at once, suggests the necessity of giving salt as a restorative. Salt is certainly serviceable in the reconstruction of the blood and tissues, after great exhaustion following disease, and during the continuance of febrile and inflammatory forms of disease. M. Berge, of Belgium, a noted chemist, has shown that common salt is essential to the healthy plasma of blood, and that the fibrin, the albumen, the musculin and the ostein, and all the protein compounds, which take a share in nutrition, become solidified, and the red globules of the blood also are dissolved. These globules are decomposed in a solution of pure albumen, whereas, an albuminous water (like the serum of the blood), containing one-hundredth part of chloride of sodium, preserves these globules without material change. In persons whose blood is deficient in saline elements there is a pallid, chorotic, and even albuminuric condition. They have but little, if any, appetite, and the gastric and salivary secretions are greatly diminished.

"In some wounded patients: or those who have undergone surgical operations, if they are fed with unsalted food, the above condition of the blood prevails. Hence, salt is essential to the wounded, as well as to those who are suffering with febrile and inflammatory diseases, and it may be also stated that the blood absorbs oxygen in direct proportion to the amount of chloride of sodium it contains, and that it stimulates also in the same proportion the chemico-vital act of nutrition, and provokes the expulsion through the kidneys, the lungs, and skin, of the nitrogenized elements of regressive nutrition of the various tissues. If these suggestions relative to the wounded and the sick were practiced, surgical operations would not be fatal in as large a per cent. of instances as they now are, nor would as many fever patients die in convalescence as now do from dropsy and debility, from imperfect nutrition. And the above consideration is not the only recommendation to physicians to give chloride of sodium to the wounded, and to those who have to submit to surgical operations, but salt should be given as an antiseptic to destroy vibrones and bacteria known to exist in the blood."

